# Effect of Electroacupuncture on Rats with Chronic Constriction Injury-Induced Neuropathic Pain

**DOI:** 10.1155/2014/129875

**Published:** 2014-01-27

**Authors:** Hsin-Cheng Hsu, Nou-Ying Tang, Yi-Wen Lin, Tsai-Chung Li, Hsu-Jan Liu, Ching-Liang Hsieh

**Affiliations:** ^1^Graduate Institute of Chinese Medicine, College of Chinese Medicine, China Medical University, 91 Hsueh-Shih Road, Taichung 40402, Taiwan; ^2^Department of Chinese Medicine, China Medical University Hospital, 2 Yuh Der Road, Taichung 40402, Taiwan; ^3^School of Chinese Medicine, College of Chinese Medicine, China Medical University, 91 Hsueh-Shih Road, Taichung 40402, Taiwan; ^4^Graduate Institute of Acupuncture Science, College of Chinese Medicine, China Medical University, 91 Hsueh-Shih Road, Taichung 40402, Taiwan; ^5^Acupuncture Research Center, China Medical University, 91 Hsueh-Shih Road, Taichung 40402, Taiwan; ^6^Graduate Institute of Biostatistics, College of Management, China Medical University, 91 Hsueh-Shih Road, Taichung 40402, Taiwan; ^7^Gradutae Institute of Integrated Medicine, College of Chinese Medicine, China Medical University, 91 Hsueh-Shih Road, Taichung 40402, Taiwan

## Abstract

We adopt the chronic constriction injury (CCI) model to induce neuropathic pain to Spragrue-Dawley (SD) rats by ligating the right sciatic nerve of using four 4-0 chromic gut sutures and subsequently applying 2 and 15 Hz electroacupuncture (EA), respectively, to the right (ipsilateral) Zusanli (St-36) and Shangjuxu (St-37) acupoints. The results of this study are summarized as follows: (1) the differences in withdrawal latencies for the radiant heat test and total lift leg counts for the cold plate test (4°C) of the control (i.e., non-EA) and sham groups were greater than those of the 2 Hz EA (2EA) and 15 Hz EA (15EA) groups; (2) the von Frey test filament gram counts of the control and sham groups were less than those of the 2EA and 15EA groups on the 6th, 7th, 8th, 11th, 12th, and 13th day following ligation; and (3) the 2EA and 15EA groups exhibited reduced cerebral transient receptor potential vanilloid type 4 (TRPV4) expressions, although we did not observe a similar effect for cerebral TRPV1 or spinal TRPV4/TRPV1 expressions. These findings show that 2 and 15 Hz EA can reduce CCI-induced neuropathic pain, which indicates that various spinal segmental and gate effects have a crucial function in pain reduction. The relationship between EA and TRPV4/TRPV1 expression requires further study.

## 1. Introduction

Previous research has shown that the prevalence rate of chronic pain in France is 31.7% and chronic pain with neuropathic characteristics is 6.9% [1], thereby showing that chronic pain with neuropathic characteristics is a health concern. Neuropathic pain is a pathological process of the somatosensory system [2], which originates from the peripheral nervous system and central nervous system (CNS) [3]. It can be caused by peripheral or central lesions, and the nature and severity of neuropathic pain are relative to the lesion [4]. Previous studies have indicated that sodium channel blocker agents such as carbamazepine and lamotrigine are a suitable treatment for intractable neuropathic pain such as trigeminal neuralgia [5, 6]. The chronic constriction injury (CCI) model is an established animal model for inducing neuropathic pain. The sciatic nerve of Sprague-Dawley (SD) rats is ligated with four 4-0 chromic gut ligatures, spaced 1 mm apart, to induce hyperalgesia, allodynia, and spontaneous pain [7]. Previous research has indicated CCI-induced neuropathic pain-related behavior is caused by the chemical toxicity of the chromic gut interacting with sciatic and sympathetic nerves, which subsequently induces peripheral or central sensitization [8]. Transient receptor potential vanilloid type 4 (TRPV4) has a crucial role in painful neuropathy such as paclitaxel-induced mechanical hyperalgesia [9]. Other researches have shown Transient receptor potential vanilloid type 1 (TRPV1) receptors to have a critical role in pain transmission and modulation in the CNS, especially in antinociceptive descending pathways such as the periaqueductal grey (PAG) and rostral ventromedial medulla [10].

Several studies have indicated that electroacupuncture (EA) activates somatostatin expression and glial cell line-derived neurotrophic factor (GDNF) proteins in the spinal dorsal horn and dorsal root ganglion, thereby modulating the analgesic effect of EA on CCI-induced neuropathic pain [11, 12]. Acupuncture and EA have been shown to regulate brain function by mediating neurotransmitters and neuropeptides in the CNS; furthermore, the induced peptide is dependent on EA frequency (e.g., 2 Hz EA induces enkephalin and *β*-endomorphin, 100 Hz EA induces dynorphin, and 15 Hz EA induces enkephalin, *β*-endomorphin, and dynorphin) [13].

Based on the discussed research, we hypothesize that EA may be suitable for the treatment of neuropathic pain. Therefore, the purpose of this study is to investigate the effect of EA on CCI-induced neuropathic pain. To achieve this, we applied 2 and 15 Hz EA to the right (ipsilateral) Zusanli (St-36) and Shangjuxu (St-37) acupoints of CCI-induced SD rats.

## 2. Materials and Methods

### 2.1. Subjects

The SD rats used in this study were pathogen-free SD male rats that weighed between 200 and 250 g (BioASCO Taiwan Co. Ltd., Taipei, Taiwan). We housed the rats under 12 h light and dark cycles in an air conditioned room (25°C) at the China Medical University Animal Center. All experimental procedures in this study were conducted in accordance with the Guide for the Care and Use of Laboratory Animals.

### 2.2. Procedure for Establishing the CCI-Induced Neuropathic Pain Model

In this study, we employed the CCI model introduced by Bennett and Xie (1988) [7] to induce neuropathic pain in SD rats. We anesthetized the rats with isoflurane gas and exposed the right sciatic nerve. We subsequently ligated the nerve proximal to the trifurcation with four 4-0 chromic gut sutures. We also exposed the left sciatic nerve, although ligation was not performed. The wounds were closed immediately using silk line before the rats were placed back in their cage.

### 2.3. Grouping

In this study, we examined 45 rats, divided randomly into the following 5 groups (9 rats per group).

1. Normal group: bilateral sciatic nerves were exposed, but no sciatic nerve ligation and EA were not performed.
2. Control group: CCI-induced neuropathic pain was established, although EA was not applied.
3. Sham group: CCI-induced neuropathic pain was established, and stainless steel acupuncture needles (Way DI MAX, Wuxi city, China; 3.4 cm in length) were inserted at the subcutaneous region (1 mm depth) of the right Zusanli (St-36) and Shangjuxu (St-37) acupoints (without manual twisting) and connected them to an EA apparatus (Trio 300, Japan). No electric stimulation was discharged.
4. 2 Hz EA (2EA) group: CCI-induced neuropathic pain was established, and stainless steel acupuncture needles were inserted into the right Zusanli (St-36) and Shangjuxu (St-37) acupoints (5 mm depth). The needles were twisted manually and connected to an EA apparatus (Trio 300, Japan). The EA frequency was set to 2 Hz.
5. 15 Hz EA (15EA) group: All tests conditions were identical to those of the 2EA group, although the EA frequency was set to 15 Hz.

### 2.4. EA Stimulation

The EA stimulation was applied on the 5th, 6th, and 7th day following ligation under isoflurane anesthesia. The EA intensity caused slight visual muscle twitching and was conducted for 20 min after completing the radiant heat, cold plate leg lift, and von Frey tests.

The radiant heat, cold plate leg lift, and von Frey tests were conducted on the 5th, 6th, 7th, 8th, 11th, 12th, and 13th day following ligation. To prevent habit learning, the tests were conducted randomly and were not performed on the 9th, and 10th day following ligation. In addition, the procedures were identical for all groups.

### 2.5. Radiant Heat Test

The radiant heat test was applied to assess the hyperalgesic behavior of the subjects. The test was performed using an apparatus (IITC Life Science Inc., USA) at 24 ± 1°C. The rats were placed in a transparent plastic box, and its window glass was an elevated flood. The rats must have adapted to the environment prior testing. The source of radiant heat was beneath the glass floor and focused at the hind paw plantar region. The timer of the test apparatus was on at the start of the simulation.

The withdrawal latency of the hind paw was measured using a stopwatch. The paw was considered withdrawn when it was raised above the flood. We measured 5 withdrawal latencies per session, with an interval of more than 5 min between any 2 tests. The irradiation time was less than 30 s per test. We calculated the average of the 5 withdrawal latencies for side, and the difference in withdrawal latency was calculated by subtracting the average withdrawal latency of the left side (control without CCI) from the average withdrawal latency of the right side (ligature with CCI). Therefore, we analyzed the differences in withdrawal latencies in this study.

### 2.6. Cold Plate Leg Lift Test at 4°C

The cold plate leg lift test at 4°C was performed to assess the spontaneous pain of the subjects. The rats were placed onto a cold hot/cold plate apparatus (Panlab, Spain). Subjects were placed in a transparent plastic box that had a floor temperature of 4°C. The total leg lift counts of the right hind paw were calculated for 5 min. Counts were excluded when the hind paw was lifted while walking.

### 2.7. Von Frey Test

The von Frey test was performed to assess allodynia using a calibrated von Frey filament (IITC Life Science Inc., USA). Subjects were placed onto a metal mesh and stimulated by a tip of the filament at the plantar region. The filament gram counts were recorded when the stimulation caused the subject to withdraw its right hind paw.

### 2.8. Western Blot Method for the Measurement of TRPV4 and TRPV1

A total of 30 rats (6 rats per group) were sacrificed on the 14th following ligation under deep anesthesia (chloral hydrate, 400 mg/kg, i.p.). We removed the brain and spinal cord (L4-6) of subjects for western blot analysis.

We immediately excised the left cerebral cortex and spinal cord (L4-6) for protein extraction. We prepared the protein by homogenizing the left cerebral cortex and spinal cords in a lysis buffer (invitrogen, USA) for 1 h at 4°C. Proteins were extracted (20 *μ*g per sample, assessed using a bicinchoninic acid protein assay) and subjected to 10% Na dodecyl sulfate-Tris glycine gel electrophoresis and transferred to a nitrocellulose membrane. The membrane was blocked using 5% nonfat milk in a Tris-buffered saline and Tween 20 (TBST) buffer (10 mmol/L Tris, pH 7.5; 100 mmol/L NaCl; 0.1% Tween 20), incubated with a primary antibody (TRPV4, TRPV1 1 : 1000 Alomone Labs) in TBST with bovine serum albumin, and incubated for 4°C overnight. A peroxidase-conjugated secondary antibody (1 : 500) was used as the secondary antibody. The membrane was developed using the ECL-Plus protein detection kit.

### 2.9. Immunohistochemistry Stain (IHC)

The remaining 15 rats (3 per group) were sacrificed under deep anesthesia (chloral hydrate, 400 mg/kg, i.p.) on the 14th day following ligation. We removed the brain and spinal cord (L4-6) of the subjects were for immunohistochemistry (IHC) staining. The subjects perfused with normal saline through the cardiac vascular system, followed by infusion with 4% paraformaldehyde (Merck, Frankfurt, Germany) in 0.1 M phosphate buffered saline (PBS, pH = 7.4). The brains and spinal cord were placed in the same fixative and stored overnight at 4°C. After being washed with PBS, the brains and spinal cords were transferred to 30% sucrose in 0.01 M PBS for cryoprotection. Subsequently, the coronal sections containing the cerebral cortex and spinal cord were cut into 20 *μ*m slices using a cryosectioning technique. The sections were preincubated (2 h at 25°C) with a mixture of 10% horse serum and 0.3% Triton X-100 in PBS to avoid nonspecific binding. Sections were subsequently incubated overnight at 4°C with a mixture of a rat monoclonal antibody against TRPV4 (1 : 1000, alomone labs) and TRPV1 (1 : 1000, alomone) in a PBS with 0.1% horse serum and 0.1% Triton X-100. Sections were subsequently incubated (2 h at 25°C) with a biotinylated-conjugated secondary antibody (1 : 200 diluted; Vector, Burlingame, CA, USA) and were further incubated with an avidin-horseradish peroxidase complex (ABC-Elite, Vector). The sections were washed 3 times with PBS between each incubation step, for 10 min per wash. Finally, the sections were visualized using 3,3′-diaminobenzidine as the chromogen. The slices were observed using a microscopy.

### 2.10. Statistical Analysis

The data are represented as mean ± standard deviation (SD). The groups were compared by one-way analysis of variance (ANOVA), followed by Tukey's test. We defined the *P* values <.05 were statistically significant.

## 3. Results

### 3.1. Effect of EA on Withdrawal Latency for the Radiant Heat Test on Rats with CCI-Induced Neuropathic Pain

For the radiant heat test, the differences in withdrawal latencies for the control, sham, 2EA, and 15EA groups were greater than those of the normal group on the 5th day following ligation (*P* < .001, Table 1), although the differences between the control and sham group latencies were similar to those between the 2EA and 15EA groups (all *P* > .05, Table 1).

The differences in withdrawal latency for the control and sham groups were greater than those of the 2EA and 15EA groups (all *P* < .001, Table 1). However, the differences between the withdrawal latencies for the control and sham groups and for the 2EA and 15EA groups were similar on the 6th, 7th, 8th, 11th, 12th, and 13th day following ligation (all *P* > .05, Table 1).

### 3.2. Effect of EA on Cold Plate Leg Lift Test in the CCI-Induced Neuropathic Pain Rat Model

For the cold plate leg lift test, the total leg lift counts for the control, sham, 2EA, and 15EA groups were greater than those of the normal group on the 5th day following ligation (all *P* < .001, Table 2), although the total leg lift counts for the control and sham groups were similar on the 5th day following ligation, as were those of the 2EA and 15EA groups (all *P* > .05, Table 2).

The total lift leg counts for the control and sham groups were greater than those of the 2EA and 15EA groups (all *P* < .001, Table 2) on the 6th, 7th, 8th, 11th, 12th, and 13th day following ligation, whereas the total lift leg counts between the control and sham groups and between the 2EA and 15EA groups were similar (all *P* > .05, Table 2).

### 3.3. Effect of EA on the Von Frey Test for the CCI-Induced Neuropathic Pain Rat Model

For the von Fey test, the filament gram counts for the control, sham, 2EA, and 15EA groups were less than those of the normal group on the 5th day following ligation (all *P* < .001, Table 3), whereas the filament gram counts for the control, sham, 2EA, and 15EA groups were similar (*P* > .05, Table 3).

The filament gram counts for the control and sham groups were less than those of the 2EA and 15EA groups (all *P* < .001, Table 3) on the 6th, 7th, 8th, 11th, 12th, and 13th day following ligation, although the filament gram counts for the control and sham groups were similar, as well as the 2EA and 15EA groups (all *P* > .05, Table 3).

### 3.4. Effect of EA on TRPV4 and TRPV1 for the CCI-Induced Neuropathic Pain Rat Model

**Figure 1:**
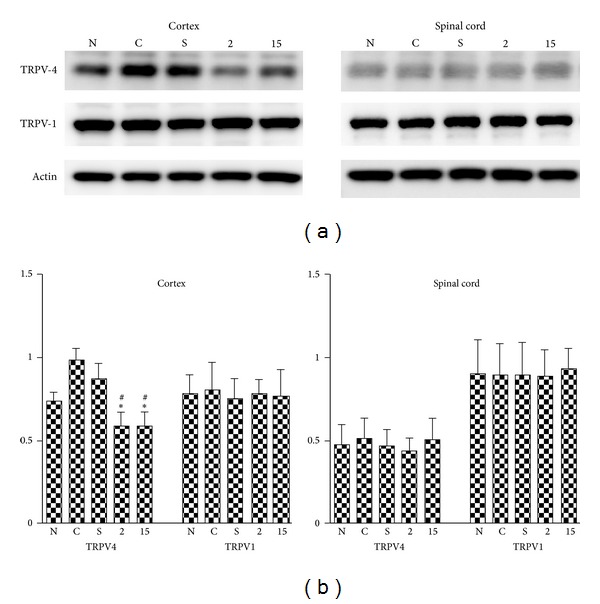
Effect of EA on TRPV4 and TRPV1 in CCI-induced neuropathic pain. TRPV4 expression in the cerebral cortex (cortex) increased in the control group without treatment (C), and these increases were reduced by 2 Hz (2) and 15 Hz (15) EA treatments at right St-36 (a) and St-37 (b) acupoints, respectively, whereas the differences in TRPV1 expressions were nonsignificant among the normal (N), C, S (sham), 2, and 15 groups (a and b). Differences in the level of TRPV4 and TRPV1 expressed in the spinal cord (Spinal cord) among the N, C, S, 2, and 15 groups (a and b) were non-significant; normal = normal group; control = control group; sham = sham group; 2EA = 2EA group; 15EA = 15EA group;*P < .01 compared to C; ^#^ *P* < .01 compared to S.

The TRPV4 levels in the cerebral cortex of the control and sham groups were greater than those of the 2EA and 15EA groups (all *P* < .01, Figures 1(a) and 1(b)). However, the difference between the cerebral cortex TRPV4 levels of the control and sham groups was non-significant (*P* > .05, Figures 1(a) and 1(b)), as well as those of the 2EA and 15EA groups (*P* > .05, Figures 1(a) and 1(b)).

The differences among the cerebral cortex TRPV1 levels (Figures 1(a) and 1(b)), spinal cord TRPV4 levels, and spinal cord TRPV1 levels (Figures 1(a) and 1(b)) were all non-significant for all groups (*P* > .05).

### 3.5. Effect of EA on TRPV4 and TRPV1 Immunostaining Positive Cells for the CCI-Induced Neuropathic Pain Rat Model

**Figure 2:**
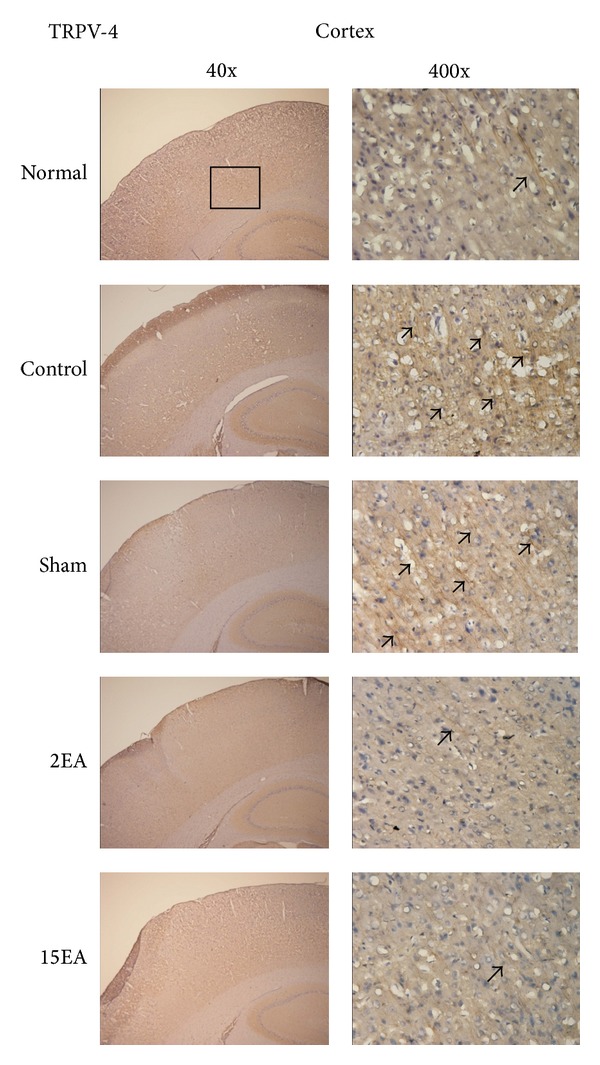
Effect of EA on cortical TRPV4 immunostaining positive cells in SD rats with CCI-induced neuropathic pain. TRPV4 immunostaining positive cells (arrow) of the cerebral cortex (cortex) increased in the control group without treatment (control) than those in the normal group (normal). These increases were reduced for the 2EA (2EA) and 15EA (15EA) treatment, but not for the Sham EA treatment (sham). normal = normal group; control = control group; sham = sham group; 2EA = 2EA group; 15EA = 15 EA group.

**Figure 3:**
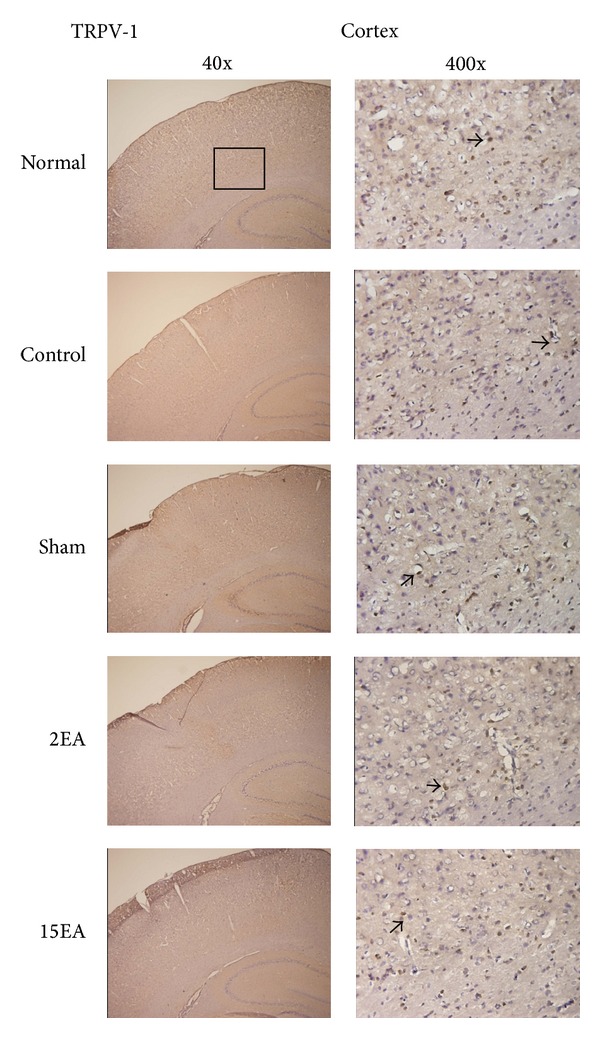
Effect of EA on cortical TRPV1 immunostaining positive cells in SD rats with CCI-induced neuropathic pain. TRPV1 immunostaining positive cells (arrow) in the cortex (Cortex) were similar among normal group (normal), control group (control), sham group (sham), 2EA group (2EA), and 15EA group (15EA).

**Figure 4:**
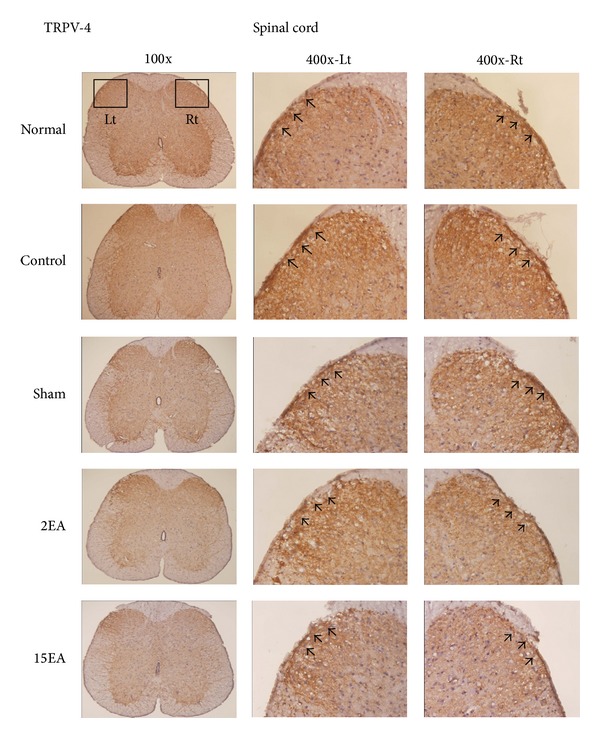
Effect of EA on spinal TRPV4 immunostaining positive cells in SD rats with CCI-induced neuropathic pain. TRPV4 immunostaining positive cells (arrow) in the spinal cord (Spinal cord) for the normal group (Normal), control group (Control), sham group (Sham), 2EA group (2EA), and 15EA group (15EA) were similar; Lt = left side of spinal cord; Rt = right side of spinal cord.

**Figure 5:**
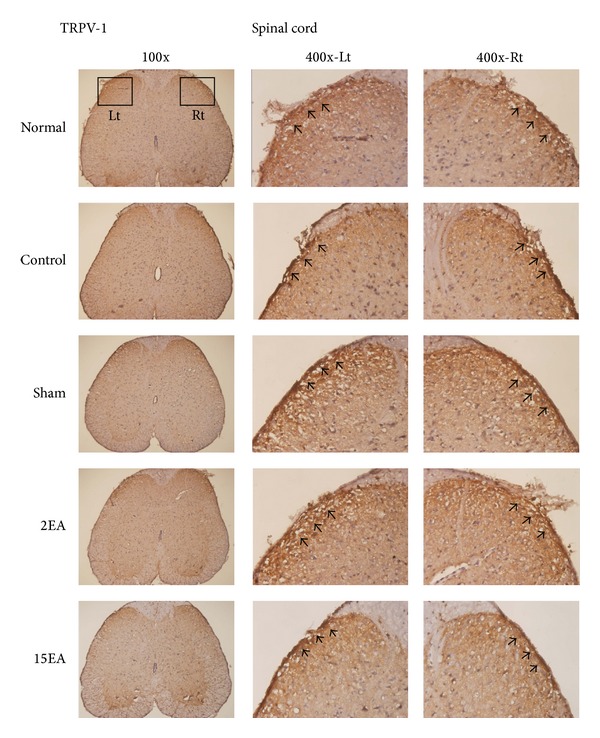
Effect of EA on spinal TRPV1 immunostaining positive cells in SD rats with CCI-induced neuropathic pain. TRPV1 immunostaining positive cells (arrow) in the spinal cord (Spinal cord) were similar among normal group (normal), control group (control), sham group (sham), 2EA group (2EA), and 15EA group (15EA); Lt= left side of spinal cord; Rt = right side of spinal cord.

The TRPV4 immunostaining positive cells in the cerebral cortex increased more prominently in the control and sham groups than those in the normal group. Furthermore, these increases reduced significantly for the 2EA and 15EA groups (Figure 2). The differences in cerebral cortex TRPV1 immunostaining positive cells for all groups were similar (Figure 3), as were those of the spinal cord TRPV4 and TRPV1 immunostaining positive cells (Figures 4 and 5).

## 4. Discussion

The results of this study show no significant difference among the control, sham, 2EA, and 15EA groups for the radiant heat test withdrawal latencies, cold plate test total leg lift counts, and von Frey test filament gram counts on the 5th day following ligation. However, these differences were greater in the control, sham, 2EA, and 15EA groups than the normal group, whereas the filament gram counts were less. Thus, the discussed CCI model for inducing neuropathic pain can be considered successful because the differences in withdrawal latencies for the radiant heat test are representative of hyperalgesic behavior [7], total lift leg counts for the cold plate test are indicative of spontaneous pain and allodynia [7], and the filament gram counts for the von Frey test are evidence of allodynia [14]. Our results also indicate that the differences in withdrawal latencies difference and total leg lift counts between the control and sham groups were greater than those between the 2EA and 15EA groups; furthermore, the von Frey test filament gram counts for the control and sham groups were less than those of the 2EA and 15EA groups on the 6th, 7th, 8th, 11th, 12th, and 13th day following ligation. Therefore, we recommend applying 2 and 15 Hz EA to the right St-36 and St-37 acupoints to relieve neuropathic pain.

Neuropathic pain can be categorized as stimulus-evoked pain, such as hyperalgesia (caused by suprathreshold noxious stimulation) or allodynia (caused by nonnoxious stimulation) and stimulus-independent pain such as spontaneous pain [15]. An ectopic discharge of myelinated A-fiber developed within 2 to 14 days following injury from injured site in CCI model of neuropathic pain, and this may contribute to the animal experiencing abnormal sensations [16]. An abnormal primary nociceptive afferent can alter central processes, thereby resulting in central sensitization [17]. Several studies have indicated that the mechanisms of neuropathic pain originate from an increase in primary afferent nociceptor firing; consequently, this may cause ectopic discharge, or because of decreases the inhibition of neuronal activity and an alternation of the central process [18]. Proinflammatory cytokines such as tumor necrosis factor-*α* (TNF-*α*) can influence long-term behavior in the CCI-induced neuropathic pain model, whereas interleukin-10 (IL-10) as an endogenous anti-inflammatory cytokine can downregulate the nerve's inflammatory reactions to the injury [19]. Pro-inflammatory cytokines such as IL-1*β*, IL-6, and TNF-*α* have been associated with the behaviors of hyperalgesia and mechanical allodynia in the CCI model of neuropathic pain [20]. The 2 Hz EA has been shown to mediate spinal mu and delta receptors, thereby relieving medical allodynia in a neuropathic pain model [14]. The 2 Hz EA has also been shown to mediate through spinal-*α*2-adrenergic and 5-HT (serotonin)_1A_, and HT_3_ reduces cold allodynia in a rat model of neuropathic pain [21]. Previous studies have shown that EA treatment enhances the expression of GDNF and the GDNF family receptor *α*-1 (GFR*α*-1) system, and raises the expression of somatostatin in the ipsilateral spinal dorsal horn, and the dorsal root ganglion relieves neuropathic pain in CCI rat models [11, 12]. Other studies have shown that 2 and 15 Hz EA can increase the release of endomorphin, enkephalin, and *β*-endorphin peptides, which involve the mu and delta receptors in the CNS [13, 22]. Previous research reported that opioid substances such as morphine are an ineffective for the treatment of neuropathic-like pain symptoms in spinal nerve ligation rat models [23], whereas applying EA to the St-36 acupoint reduces pain behavior and excitability of the ipsilateral dorsal horn in CCI-induced neuropathic pain rat models [24], and the antinociceptive effect of acupuncture results from the activation of adenosine A1 receptor expression situated on the ascending nerve [25]. The 2 and 15 Hz may mediate via increasing TRPV-1 positive neurons and central terminals of the spinal dorsal horn to reduce damage induced by resiniferatoxin in rat [26]. Furthermore, the spinal segmental and supraspinal effect is involved in relieving pain of spinal cord stimulation in a rat model of neuropathic pain [27], and EA has also been shown to mediate through the gate control to reduce neuropathic pain [28]. Overall, we recommend applying 2 and 15 Hz to the right St-36 and St-37 acupoints to relieve CCI-induced neuropathic pain, the effect of which originates from various including endogenous opioid, somatostatin, adrenalin and serotonin peptides, GDNF, adenosine A1 receptor expression, and the effect of spinal segmental and gate effect. The generation of the segmental and gate effects has a critical role because the right sciatic nerve and right St-36 and St-37 acupoints are located in an identical spinal nerve segment.

Our results also indicate that ipsilateral 2 and 15 Hz EA may reduce cerebral TRPV4 expressions, although a similar effect was not observed for cerebral TRPV1, spinal TRPV4, or spinal TRPV1. TRPV4 functions as an osmoreceptor because it is sensitive to hypoosmotic swelling [29]. TRPV4 is also involved in the osmolar detection of nociceptor enhancing the production of prostaglandin E2 that is a hyperalgesic inflammatory mediator; thus, the role of TRPV4 is diverse and also is a pain signaling transducer [30]. Previous research has shown that TRPV4 expression has a critical function in paclitaxel-induced neuropathic models because a decrease in TRPV4 expression reduces paclitaxel-induced nociception pain behavior [9]. Other studies have shown that TRPV1 is a nonselective cation channel for sensory nerves, and the expression of TRPV1 can be induced by heat and capsaicin [31, 32]. TRPV1 also has a crucial function in afferent of experimental arthritis by triggering peripheral neuropeptides release, such as calcitonin-gene related peptide [31]. The stimulation of TRPV1 receptors in PAG can activate the production of analgesia in antinociceptive descending pathways [10]. Furthermore, TRVP1 has been shown to be sensitized by inflammatory heat stimulation [32, 33]. Based on this discussion, we assert that cerebral TRPV4 is easier to affect than spinal TRPV4 including pain signals and osmotic. Cerebral and spinal TRPV1 primarily respond to inflammation and heat stimulation, and this effect of EA on inflammation and heat stimulation needs further study.

Based on the results of this study, we recommend applying 2 and 15 Hz EA to the right St-36 and St-37 acupoints to reduce CCI-induced neuropathic pain in rat models. This effect of EA suggests that results from various mechanisms but spinal segmental and gate effects play an important role. The relationship between EA and TRPV4/TRPV1 requires further study.

## Figures and Tables

**Table 1:** Effect of electroacupuncture (EA) on radiant heat test on SD rat with chronic constriction injury (CCI)-induced neuropathic pain (sec.).

	Normal	Control	Sham	2EA	15EA
D5	−0.01 ± 0.45	7.72 ± 0.54*	8.10 ± 1.02*	7.70 ± 0.87*	8.12 ± 0.66*
D6	0.01 ± 0.41	7.42 ± 1.22*	6.88 ± 1.35*	2.57 ± 0.85^∗#¶^	3.85 ± 0.66^∗#¶^
D7	0.08 ± 0.69	7.91 ± 1.66*	6.50 ± 1.07*	2.41 ± 0.71^∗#¶^	3.00 ± 0.85^∗#¶^
D8	0.16 ± 0.38	8.68 ± 1.17*	6.94 ± 0.94*	2.00 ± 0.65^∗#¶^	3.04 ± 1.23^∗#¶^
D11	−0.06 ± 0.44	8.24 ± 1.40*	7.72 ± 1.79*	1.87 ± 1.24^∗#¶^	1.93 ± 0.79^∗#¶^
D12	−0.11 ± 0.65	7.59 ± 1.41*	6.70 ± 0.90*	1.71 ± 0.65^∗#¶^	1.92 ± 1.04^∗#¶^
D13	0.17 ± 0.65	7.02 ± 0.85*	7.18 ± 0.98*	1.90 ± 1.12^∗#¶^	2.52 ± 0.69^∗#¶^

Data expressed as mean ± standard deviation. *N* = 9; normal: normal group, no CCI, no EA; control: control group, CCI without EA; sham: sham group, CCI with sham EA; 2EA: 2EA group, CCI with 2 Hz EA on ipsilateral Zusanli andShangjuxu acupoints; 15EA: 15EA group, CCI with 15 Hz EA on ipsilateral Zusanli and Shangjuxu acupoints; D5: 5th day following ligation; D6: 6th day following ligation; D7: 7th day following ligation; D8: 8th day following ligation; D11: 11th day following ligation; D12: 12th day following ligation; D13: 13th day following ligation; *P < .001 compared with normal'; ^#^ *P* < .001 compared with Control;  ^¶^ *P* < .001 compared with sham.

**Table 2:** Effect of electroacupuncture (EA) on cold plate lift leg test at 4°C in SD rats with chronic constriction injury (CCI)-induced neuropathic pain (counts).

	Normal	Control	Sham	2EA	15EA
D5	0.22 ± 0.67	24.67 ± 5.27*	26.00 ± 4.74*	22.89 ± 3.10*	26.11 ± 3.76*
D6	0.11 ± 0.33	27.78 ± 8.74*	24.78 ± 7.22*	15.22 ± 3.70^∗#¶^	13.56 ± 5.13^∗#¶^
D7	0.11 ± 0.33	26.00 ± 8.53*	25.78 ± 7.79*	13.22 ± 6.57^∗#¶^	14.33 ± 4.85^∗#¶^
D8	0.00 ± 0.00	27000 ± 8.44*	26.33 ± 6.61*	13.56 ± 4.10^∗#¶^	14.44 ± 3.24^∗#¶^
D11	0.11 ± 0.33	32.11 ± 7.83*	29.56 ± 8.65*	16.89 ± 7.29^∗#¶^	16.67 ± 7.33^∗#¶^
D12	0.00 ± 0.00	32.33 ± 6.82*	32.00 ± 9.34*	14.44 ± 6.42^∗#¶^	14.67 ± 6.40^∗#¶^
D13	0.00 ± 0.00	29.33 ± 6.60*	30.89 ± 10.18*	14.78 ± 8.24^∗#¶^	17.44 ± 7.84^∗#¶^

Data represent mean ± standard deviation. *N* = 9; normal: normal group, no CCI, no EA; control: control group, CCI without EA; sham: sham group, CCI with sham EA; 2EA: 2EA group, CCI with 2 Hz EA on ipsilateral Zusanli and Shangjuxu acupoints; 15EA: 15EA group, CCI with 15 Hz EA on ipsilateral Zusanli and Shangjuxu acupoints; D5: 5th day following ligation; D6: 6th day following ligation; D7: 7th day following ligation; D8: 8th day following ligation; D11: 11th day following ligation; D12: 12th day following ligation; D13: 13th day following ligation; *P < .001 compared with normal'; ^#^ *P* < 0.001 compared with control; ^¶^ *P* < .001 compared with sham.

**Table 3:** Effect of electroacupuncture (EA) on von Frey test in SD rats with chronic constriction injury (CCI)-induced neuropathic pain (counts).

	Normal	Control	Sham	2EA	15EA
D5	39.76 ± 1.58	11.82 ± 1.76*	11.33 ± 1.08*	11.82 ± 3.48*	11.23 ± 0.91*
D6	36.50 ± 6.54	10.93 ± 4.12*	12.70 ± 2.00*	20.07 ± 1.74^∗#¶^	18.48 ± 2.42^∗#¶^
D7	40.51 ± 2.10	11.21 ± 3.84*	12.97 ± 2.65*	18.59 ± 2.16^∗#¶^	18.13 ± 1.58^∗#¶^
D8	40.54 ± 3.01	9.91 ± 2.48*	10.97 ± 2.03*	18.53 ± 2.29^∗#¶^	17.94 ± 1.90^∗#¶^
D11	40.23 ± 4.99	9.38 ± 3.36*	9.26 ± 1.95*	18.87 ± 2.33^∗#¶^	17.94 ± 1.93^∗#¶^
D12	41.59 ± 3.01	8.86 ± 2.14*	10.03 ± 2.15*	20.37 ± 3.29^∗#¶^	19.72 ± 1.76^∗#¶^
D13	41.26 ± 5.13	7.99 ± 2.17*	8.26 ± 1.70*	17.81 ± 2.79^∗#¶^	20.14 ± 1.65^∗#¶^

Data represent mean ± standard deviation. *N* = 9; normal: normal group, no CCI, no EA; control: control group, CCI without EA; Sham: sham group, CCI with sham EA; 2EA: 2EA group, CCI with 2 Hz EA on ipsilateral Zusanli and Shangjuxu acupoints; 15EA: 15EA group, CCI with 15 Hz EA on ipsilateral Zusanli and Shangjuxu acupoints; D5: 5th day following ligation; D6: 6th day following ligation; D7: 7th day after operation; D8: 8th day following ligation; D11: 11th day following ligation; D12: 12th day following ligation; D13: 13th day following ligation; *P < .001 compared with normal; ^#^ *P* < .001 compared with control; ^¶^ *P* < 0.001 compared with sham.
